# ALDH1A1 Deficiency in Gorlin Syndrome Suggests a Central Role for Retinoic Acid and ATM Deficits in Radiation Carcinogenesis

**DOI:** 10.3390/proteomes2030451

**Published:** 2014-09-11

**Authors:** Thomas J. Weber, Thierry Magnaldo, Yijia Xiong

**Affiliations:** 1 Systems Toxicology and Exposure Science, Pacific Northwest National Laboratory, Richland, WA 99352, USA; 2 Faculté de Médicine, 2ème étage, CNRS UMR 6267—INSERM U998—UNSA, Nice 06107 Cedex 2, France; E-Mail: Thierry.Magnaldo@unice.fr; 3 College of Osteopathic Medicine of the Pacific-Northwest, Western University of Health Sciences, Lebanon, OR 97355, USA; E-Mail: yxiong@westernu.edu

**Keywords:** Gorlin, ATM, carcinogenesis, retinoic acid

## Abstract

We hypothesize that aldehyde dehydrogenase 1A1 (ALDH1A1) deficiency will result in impaired ataxia-telangiectasia mutated (ATM) activation in a retinoic acid-sensitive fashion. Data supporting this hypothesis include (1) reduced ATM activation in irradiated primary dermal fibroblasts from ALDH1A1-deficient Gorlin syndrome patients (GDFs), relative to ALDH1A1-positive normal human dermal fibroblasts (NHDFs) and (2) increased ATM activation by X-radiation in GDFs pretreated with retinoic acid, however, the impact of donor variability on ATM activation in fibroblasts was not assessed and is a prudent consideration in future studies. Clonogenic survival of irradiated cells showed differential responses to retinoic acid as a function of treatment time. Long-term (5 Day) retinoic acid treatment functioned as a radiosensitizer and was associated with downregulation of ATM protein levels. Short-term (7 h) retinoic acid treatment showed a trend toward increased survival of irradiated cells and did not downregulate ATM protein levels. Using a newly developed IncubATR technology, which defines changes in bulk chemical bond patterns in live cells, we can discriminate between the NHDF and GDF phenotypes, but treatment of GDFs with retinoic acid does not induce reversion of bulk chemical bond patterns associated with GDFs toward the NHDF phenotype. Collectively, our preliminary investigation of the Gorlin phenotype has identified deficient ALDH1A1 expression associated with deficient ATM activation as a possible susceptibility factor that is consistent with the high incidence of spontaneous and radiation-induced carcinogenesis in these patients. The IncubATR technology exhibits sufficient sensitivity to detect phenotypic differences in live cells that may be relevant to radiation health effects.

## 1. Introduction

We are investigating biochemical mechanisms underlying pathophysiological aspects of Gorlin syndrome in an effort to improve our understanding of molecular risk factors for radiation carcinogenesis. Gorlin syndrome is an autosomal dominant disease associated with developmental abnormalities and a dramatic predisposition to spontaneous and radiation-induced cancers [1]. Therefore, cells from Gorlin syndrome patients contain a blueprint for aberrant molecular features of importance to radiation carcinogenesis. Research projects have focused on germline mutations in the *Patched* gene in Gorlin syndrome, which is hypothesized to render these patients haploinsufficient. The *Patched* gene codes for a 12-span transmembrane receptor (PTCH) for hedgehog ligands that is believed to function as a tumor suppressor [2]. In the absence of Hedgehog ligands PTCH inhibits the activity of a second transmembrane protein termed SMOOTHENED, and binding of Hedgehog ligands to PTCH inhibits this repression, resulting in the activation of a signaling cascade whose output function is mediated by GLI transcription factors [2]. Dysfunctional regulation of the Hedgehog pathway is implicated in both developmental abnormalities and carcinogenesis, which are primary pathophysiological features of Gorlin syndrome.

We have interrogated primary cells from Gorlin syndrome patients using activity-based proteomics [3]. Results from our proteomics study demonstrated that aldehyde dehydrogenase 1A1 (ALDH1A1) protein expression was deficient in primary dermal fibroblasts from Gorlin syndrome patients (GDFs), as compared with primary normal human dermal fibroblasts (NHDFs) used as controls. Further, two different Gorlin syndrome donors characterized by distinct *Patched* mutations displayed loss of ALDH1A1 protein. These observations have provided a model system analogous to loss-of-function where acute molecular effects of radiation can be compared in ALDH1A1 positive NHDFs *vs.* ALDH1A1 negative GDFs. ALDH1A1 is one of three rate-limiting enzymes that convert retinaldehyde to retinoic acid. Retinoic acid synthesis is decreased by 77% in the Aldh1a1 knockout mouse [4], indicating that ALDH1A1 accounts for a major fraction of retinoic acid physiologically. Therefore, ALDH1A1 deficiency suggests that Gorlin syndrome patients may display retinoic acid-deficiency. Retinoic acid is pivotal in developmental biology [5] and retinoic acid deficiency increases cancer risk broadly [6,7,8,9]. Therefore, retinoic acid deficiency can impact the same pathophysiological features of Gorlin syndrome (developmental abnormalities and carcinogenesis) attributed to the Hedgehog pathway. In this context, the Hedgehog and retinoic acid pathways are intertwined [8] making it difficult to dissect their individual roles in pathophysiology. Specific cellular and molecular processes that are regulated by retinoic acid that can impact radiation carcinogenesis include DNA damage repair [10,11] and regulation of the stem cell niche [12,13]. Alternatively, ALDH1A1 has many additional physiological functions, including the regulation of adipogenesis, glucose tolerance, suppression of thermogenesis, induction of oncogene suppressors and xenobiotic metabolism [14,15] that could also impact carcinogenesis. The identification of retinoid-specific molecular processes may provide opportunities to investigate mechanisms of action that could aid in dissecting the role of these pathways in radiation carcinogenesis.

Deficient DNA damage repair is an established risk factor for radiation carcinogenesis [16] and retinoic acid may regulate the activity of the ATM kinase [10]. Studies were undertaken to determine whether ALDH1A1 deficiency, which is hypothesized to result in a retinoic acid-deficient phenotype, is associated with diminished ATM activation in GDFs, relative to NHDFs. Subsequently, we investigated how retinoic acid treatment impacted clonogenic survival of GDFs and applied a new omic technology that can define changes in bulk chemical bond patterns in live cells to determine whether the NHDF and GDF phenotypes with and without retinoic acid treatment could be discerned.

## 2. Experimental

### 2.1. Cell Culture

NHDFs were purchased from Lonza (51 years old donor, Allendale, NJ, USA) and were used as controls. GDFs were obtained with informed written consent of patients [17]. GDFs (cell line identifier—AS573; 45 year old donor) used in the present study were isolated from healthy photo-shielded skin of Gorlin syndrome patients and the *Patched* mutation for these cells is defined [18]. Fibroblasts were maintained in RPMI supplemented with 10% FBS, 1 ng/mL bFGF, 2 mM Glutamax, 100 U/mL penicillin, 100 mg/mL streptomycin, 25 mg/mL amphotericin B in 5% CO_2_/95% air at 37 °C, and were subcultured by trypsinization. A single cell line was used to interrogate the NHDF and GDF phenotype in our initial studies to exploit the loss of ALDH1A1 protein levels in GDFs in comparison to the ALDH1A1 positive NHDFs. It will be prudent in future studies to determine whether donor variability also has significant impact on retinoic acid-sensitive ATM activation. In some experiments cells were treated with the ATM kinase inhibitor KU-55933 (20 nM final; Selleck Chemicals, Houston, TX, USA) or all-trans retinoic acid (Sigma-Aldrich, St. Louis, MO, USA). For experiments investigating ATM activation, cells were maintained until 2 days postconfluent at which time they were serum-starved for 24 h prior to retinoic acid treatment and/or irradiation to define the acute DNA damage response and its sensitivity to retinoic acid. For long-term retinoic acid treatment (5 day) medium supplemented with DMSO or retinoic acid was changed daily.

### 2.2. Irradiation

Fibroblasts were exposed to 1–2 Gy X-radiation (0.58 Gy/min; 2 keV·μm^−1^) using a Therapax X-RAD 320 system equipped with 320 kV high stability X-ray generator (operated at 300 kV for cell irradiations), metal ceramic X-ray tube, variable X-ray beam collimator and #8 filter (Precision X-ray Incorporated, East Haven, CT, USA).

### 2.3. Clonogenic Survival

For clonogenic survival assays, 1 × 10^4^ cells were seeded into 10 cm dishes and allowed to attach for 24 h. Cells were then irradiated and immediately after irradiation the medium was aspirated, the cells were overlaid with a permissive agarose (6 mL of a 0.8% agar solution, [19]) and fresh growth medium was added. The agarose reduces (but does not fully inhibit) fibroblast migration, thereby improving the definition of colony edges and quantification. The dishes were then wrapped in parafilm and colonies were quantified on day 10 as previously described [3]. The agar layer is removed by inverting the dish and dislodging the agar layer with gentle tapping prior to staining with crystal violet. Visual examination of the agar plug did not reveal evidence that colonies had stuck to the agar and were removed from the plate upon inversion.

### 2.4. IncubATR

We have recently developed a new “omics” technology (termed the IncubATR) which detects changes in the vibrational-rotational structure of chemical bonds in live cells using attenuated total reflectance-Fourier transform infrared (ATR-FTIR) spectrometry [20]. The IncubATR detects global changes in bulk chemical bond patterns in a non-destructive and unbiased fashion. Information about the cells proteome is captured within the bulk chemical bond patterns specifically in spectral regions that encompass protein fingerprints (Amide bands I and II). Cells are seeded on an ATR crystal (ZnSe) with fibronectin (10 ng/mL) included in the medium to aid in cell attachment. The IncubATR chamber (Simplex Scientific, Middleton, WI, USA) fits inside the FTIR and has temperature and gas control to maintain cell viability. Chemical bond information is captured from an evanescent wave that extends into the cell monolayer at multiple points throughout the crystal providing a representative sampling of the cell system. Automated FTIR data collection was accomplished using the Macros basic script program to control the spectrometer (Thermo Nicolet 4700 FTIR; Thermo Scientific, Madison, WI, USA). Cell culture medium spiked with fibronectin was used as background for subtraction to provide the cell-specific signal. The program collected one FTIR spectrum every 15 min for approximately 8 h. The spectrum was collected at 4 cm^−1^ resolution with automatic atmospheric suppression and 512 scans were averaged for each spectrum. Since IncubATR technology is based on the ATR principle, ATR correction was applied to all spectra using the software from Thermo Nicolet before further analysis.

### 2.5. Principle Component Analysis

Principle component analysis has been used to analyze FTIR data and to differentiate between sample groups. Each set of FTIR data collected by IncubATR technology contained about 30 FTIR spectra. Those data were exported as ASCII files and spectra from all three cell types were put together and imported into the statistical computing software R (freeware version 3.0.2; [21]) for further data manipulation and principle component analysis. Note that in a complex system, FTIR peaks may not just change intensity but also shift position along the wavenumber axis and it is preferable to do the principle component analysis on the whole spectrum as opposed to selected peak features. There was significant noise at both ends of the FTIR spectra (<850 cm^−1^ or >3150 cm^−1^). These spectral ranges were removed before subjecting data sets to principle component analysis. The spectral range of 2250–2450 cm^−1^ contained the absorption peaks of carbon dioxide which is not relevant to the cell’s FTIR signal [22] and was also removed before analysis. The processed spectra were then subjected to principle component analysis. Detailed assignments can be found in [23]. Spectral data were centered (*i.e.*, subtracted means at all wavenumbers) but not scaled (*i.e.*, not normalized by standard deviation) and the relative intensity of the spectral peaks was taken into consideration during the principle component analysis.

### 2.6. Western Blot Analysis

Indices of ATM activation were determined by Western blot using previously described methods [24]. Final titers were: ATM (1:3000; Cell Signaling Technologies; Beverly, MA, USA), P-ATM (1:2000; Cell Signaling Technologies; Beverly, MA, USA), SP1 (1:4000; Santa Cruz Biotechnology, Dallas, TX, USA), P-SP1 (1:3000; Active Motif, Carlsbad, CA, USA), actin (1:10,000; EMD Millipore; Billerica, MA, USA), DNA-PKcs (1:4000; Santa Cruz Biotechnology, Dallas, TX, USA), secondary antibodies (1:3000; Thermo Scientific, Rockford, IL, USA).

### 2.7. Statistics

For Western blots and clonogenic survival assays, individual comparisons were made using the Students t-test or ANOVA with a post hoc Student’s Newman-Keul test, as appropriate. The *p* < 0.05 level was accepted as significant.

## 3. Results and Discussion

**Figure 1:**
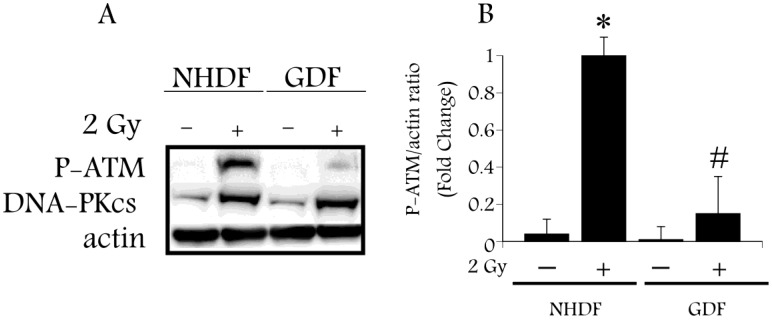
Western blot analysis of ATM activation by 2 Gy X-ray in Gorlin syndrome patients (GDFs) compared with normal human dermal fibroblasts (NHDFs). **Panel A**: Western blot showing anti-phospho-ATM antibody (P-ATM) and DNA-PKcs protein levels following exposure of NHDFs and GDFs to 2 Gy X-radiation at 10 min post-exposure; **Panel B**: Quantification of western blot results. Graph represents the pooled results from two separate experiments performed in duplicate (pooled results, *n* = 4) with values expressed as the mean ± SE. * Significantly different from NHDF sham control, *p* < 0.05. ^#^ Significantly different from 2 Gy NHDF group, *p* < 0.05.

Radiation-induced DNA damage regulates ATM activation, an event associated with phosphorylation of ATM on S^1981^ [25]. Upon activation ATM phosphorylates a number of substrates, including the SP1 transcription factor at S^101^ [26]. ATM activation is sensitive to retinoic acid [10], and therefore provides a focal point that is relevant to both radiation-induced DNA damage and retinoic acid signaling. We hypothesize that retinoic acid deficiency, as a consequence of ALDH1A1 deficiency, will impair ATM activation in GDFs, as compared with ATM activation in NHDFs. Western blot analysis using anti-phospho-ATM antibody (P-ATM; S^1981^) detected a significant increase in P-ATM levels 10 min after exposure of NHDFs to 2 Gy X-radiation, as compared with diminished P-ATM levels in irradiated GDFs (Figure 1A,B), supporting this hypothesis. In contrast, protein levels of the DNA-dependent protein kinase catalytic subunit (DNA-PKcs) induced by radiation were increased to a comparable extent in both GDFs and NHDFs, suggesting that ALDH1A1 deficiency specifically impacted ATM activation.

**Figure 2:**
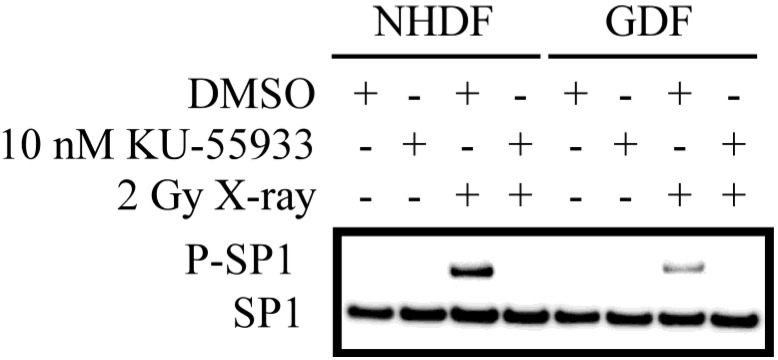
Western blot analysis of ATM-dependent phosphorylation of SP1. Western blot for P-SP1 in cells exposed to 2 Gy X-radiation at 10 min post-exposure. Similar results were observed in two separate experiments.

As an additional read-out for ATM activity, we defined the ATM-dependent phosphorylation of SP1 (S^101^; Figure 2). P-SP1 levels show a marked increase following irradiation of NHDFs. While P-SP1 levels are also increased in irradiated GDFs, the relative levels are reduced by ≈70%) as compared to NHDFs. Dependence of SP1 phosphorylation on ATM kinase activity was confirmed by pretreatment of cells with the ATM inhibitor KU-55933 [27], which eliminated P-SP1 levels in NHDFs and GDFs. This observation demonstrates that decreased ATM activation (Figure 1) translates to decreased phosphorylation of an established ATM substrate.

**Figure 3:**
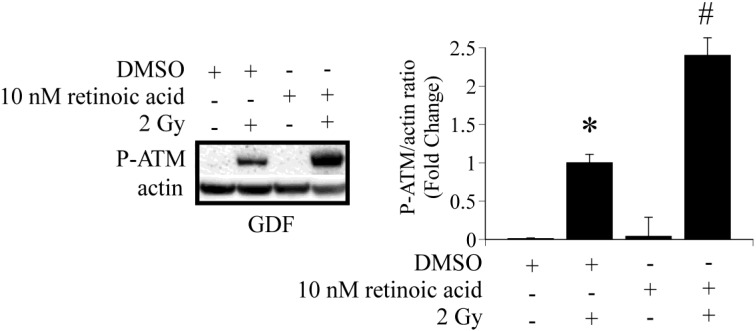
Retinoic acid pre-treatment enhances radiation-dependent ATM activation in GDFs. GDFs were exposed to 2 Gy X-radiation with or without pretreatment for 60 min with 10 nM retinoic acid. P-ATM levels were determined 10 min post-exposure. Graph represents the pooled results from two separate experiments with values expressed as the mean ± SE (*n* = 4). * Significantly different from GDF sham control, *p* < 0.05. ^#^ Significantly different from 2 Gy NHDF group, *p* < 0.05.

Studies were conducted to determine if it is possible to increase ATM activation in GDFs by addition of retinoic acid to culture medium. GDFs were pretreated with 10 nM retinoic acid for 60 min, exposed to 2 Gy X-radiation and P-ATM levels were determined 10 min post-exposure by western blot. Retinoic acid pretreatment significantly increased radiation-dependent P-ATM protein levels, compared with irradiated DMSO pretreated controls (Figure 3). This observation confirms that ATM activation is sensitive to retinoic acid.

**Figure 4:**
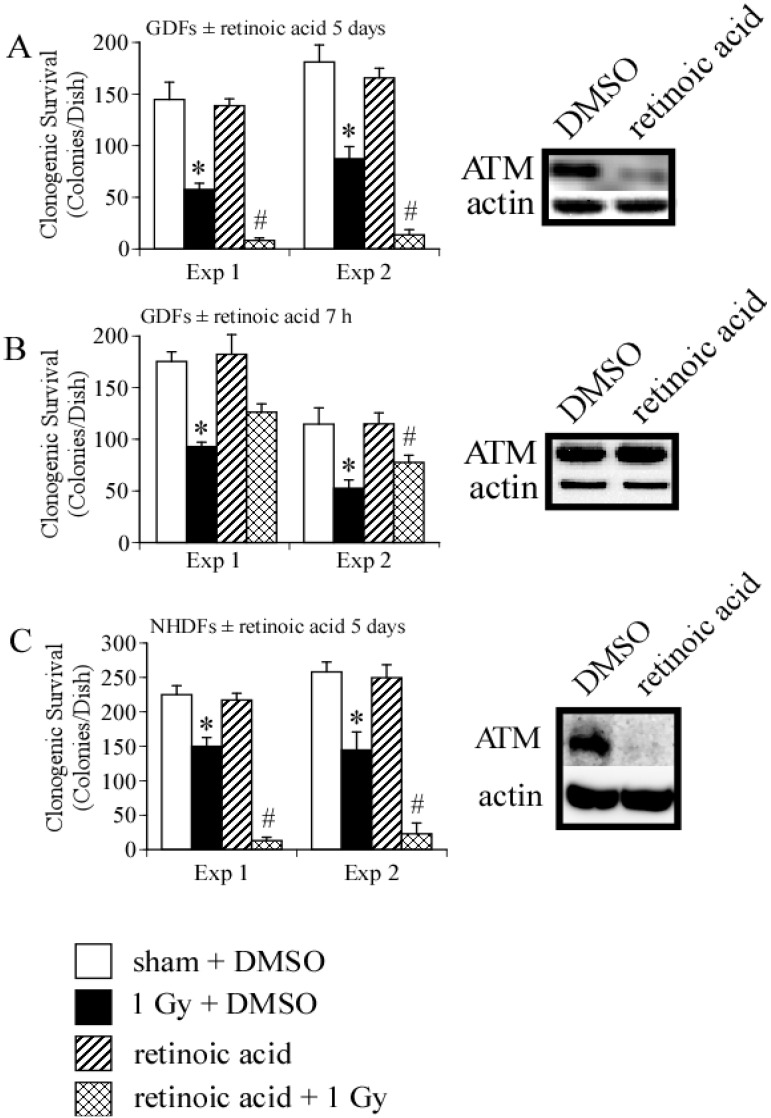
Retinoic acid treatment modulates clonogenic survival and ATM protein levels. **Panel A**: Clonogenic survival and qualitative assessment of ATM protein levels for GDFs maintained in medium supplemented with 10 nM retinoic acid for 5 days prior to irradiation. For panels A–C: (1) Results of two independent clonogenic survival assays are shown (Exp 1, Exp 2) and (2) experimental groups include sham + DMSO (white), 1 Gy X-radiation + DMSO (black), retinoic acid (hatch), 1 Gy X-radiation + retinoic acid (crosshatch); **Panel B**: Clonogenic survival and qualitative assessment of ATM protein levels for GDFs maintained in medium supplemented with DMSO or 10 nM retinoic acid for 1 h before exposure to 1 Gy X-ray with retinoic acid maintained in medium for 6 h after irradiation; **Panel C**: Clonogenic survival and ATM protein levels for NHDFs maintained in medium supplemented with 10 nM retinoic acid for 5 days prior to irradiation. All values represent the mean ± SE (*n* = 3). * Significantly different from sham control, *p* < 0.05. ^#^ Significantly different from 1 Gy, *p* < 0.05.

We then asked whether long- or short-term treatment of GDFs with retinoic acid modulated clonogenic survival following exposure to 1 Gy X-radiation. We have previously demonstrated that 1 Gy is in the linear range for clonogenic survival using these specific cells [3], therefore, this dose enables detection of positive or negative changes in clonogenic survival induced by retinoic acid. We initially examined clonogenic survival in GDFs maintained in medium supplemented with 10 nM retinoic acid for 5 days. Figure 4A demonstrates that retinoic acid pretreatment significantly reduced clonogenic survival of GDFs following 1 Gy X-ray exposure, as compared with irradiated DMSO-treated controls, suggesting that long-term retinoic acid treatment functioned as a radiosensitizer. Under this condition, ATM protein levels were markedly reduced (see western blot Panel A). Figure 4B illustrates the results for GDFs maintained in medium supplemented with 10 nM retinoic acid 1 h before and maintained in medium supplemented with retinoic acid for 6 h after irradiation (7 h total). At 6 h post-irradiation, cells were washed with normal growth medium to remove retinoic acid, the cells were then overlaid with permissive agarose and clonogenic survival was determined as described in Methods. Retinoic acid treatment resulted in a trend toward increased survival of GDFs, however, the effect was at the borderline of statistical significance (significant in one of two experiments with trend apparent in both experiments). We have not yet investigated whether retinoic acid dose or related pharmacons (e.g., 9-*cis* retinoic acid) can be optimized to improve this response further. In the short-term treatment group, retinoic acid did not downregulate ATM protein levels (determined 6 h post irradiation, see western blot Panel B). It is established that retinoic acid can function as a radiosensitizer [28], therefore, we asked whether retinoic acid-mediated radiosensitization under the conditions employed was unique to GDFs. Treatment of NHDFs with 10 nM retinoic acid for 5 days resulted in significant radiosensitization and downregulation of ATM protein levels (Figure 4C). These observations highlight a potential role for ATM downregulation in retinoic acid-mediated radiosensitization and suggest that retinoic acid treatment time is an important consideration when examining ATM activation.

**Figure 5:**
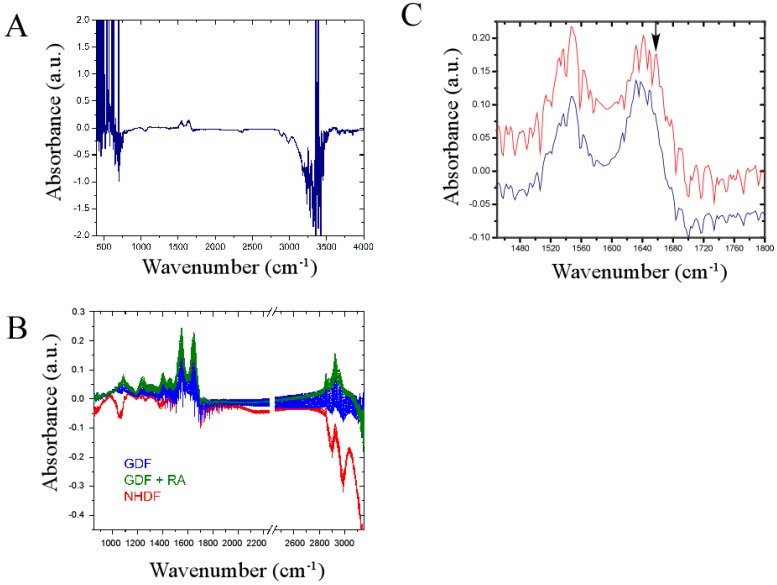
Infrared spectral signature of NHDFs and GDFs in live cell studies. **Panel A**: Raw spectrum illustrating regions of high noise (<850 cm^−1^ or >3150 cm^−1^); **Panel B**: Primary spectroscopic region used for principle component analysis with omission of regions encompassing high noise and the carbon dioxide signal (2250–2450 cm^−1^); **Panel C**: Magnified view of spectrum encompassing Amide bands I and II showing highly conserved and differential (see arrow) peaks when NHDFs (red) and GDFs (blue) are compared. a.u. = arbitrary units.

We investigated whether a newly developed omics technology (termed the IncubATR) had sufficient sensitivity to discriminate bulk chemical bond patterns in live cell studies comparing (1) NHDFs *vs.* GDFs and (2) GDFs *vs.* GDFs + retinoic acid as described in Methods. To illustrate how data are processed, Figure 5A shows the raw spectrum for NHDFs. Figure 5C illustrates a magnified view of the spectral region containing Amide bands I and II to show the spectral fingerprints for NHDFs (red) and GDFs (blue) in greater detail. Observable peaks are qualitatively very similar between experimental groups, indicating that many of the spectral features detected are conserved (*i.e.*, fibroblasts from 2 different donors showed high similarity). However, differences in the presence or absence of some peaks were noted (see arrow as one example). In the example highlighted by the arrow, the indicated band correlates to membrane-associated helical proteins based on infrared spectroscopy correlation tables. Whether this assignment is true for bulk chemical bond patterns in live cells has yet to be determined. We subsequently used principle component analysis to determine whether sufficient differences were present to discriminate between experimental groups.

**Figure 6:**
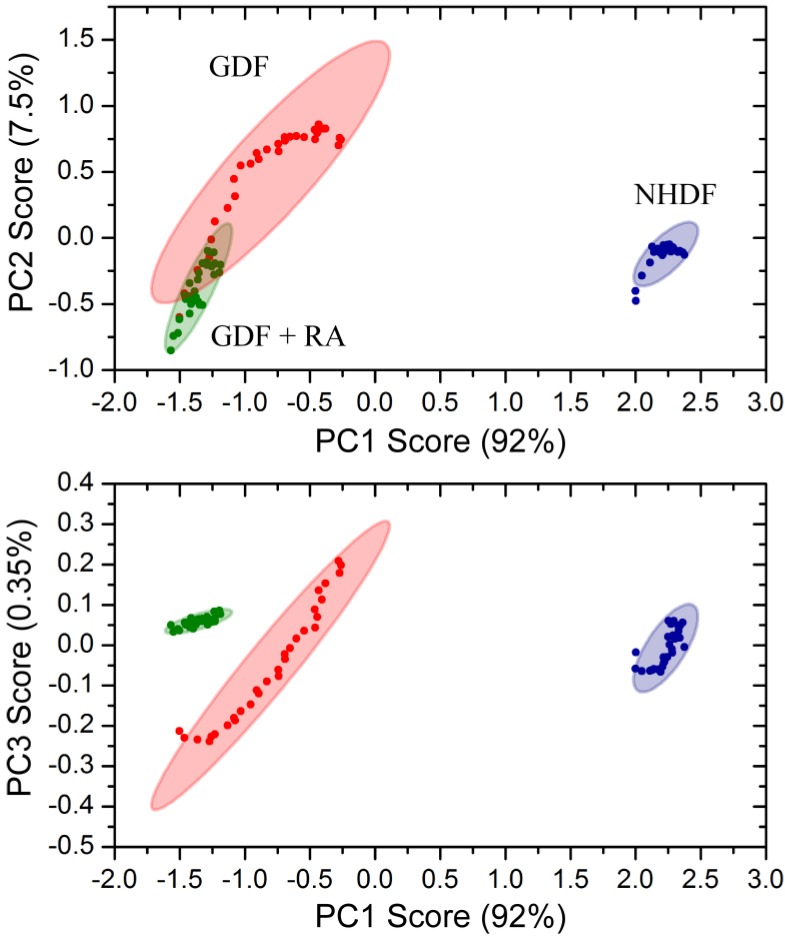
Principle component analysis of bulk chemical bond patterns in NHDFs and GDFs. Principle component analysis exhibited sufficient sensitivity to discriminate between (1) NHDFs *vs.* GDFs and (2) GDFs *vs.* GDFs + 10 nM retinoic acid for 5 days.

Principle component analysis of bulk chemical bond patterns in NHDFs and GDFs demonstrated that there are sufficient differences in the spectra to discriminate between experimental groups (Figure 6). Principle component analysis could further discriminate GDFs from GDFs that were maintained in medium supplemented with 10 nM retinoic acid for 5 days. The proportions of variance explained by the first 3 principle components were 91.76%, 7.54% and 0.35% and their cumulative proportion was 99.65%. Thus, the first three principle components explained more than 99.6% of the variance in the spectra. Under this condition, retinoic acid treatment (5 day) functions as a radiosensitizer (see Figure 4). Thus, the IncubATR technology has the sensitivity to detect phenotypic differences associated with the radiosensitive phenotype (GDFs), as well as perturbation by retinoic acid, and future studies will begin determining whether the underlying features can be resolved mechanistically.

## 4. Conclusions

We have demonstrated that ALDH1A1 deficiency in cells from Gorlin syndrome patients is associated with (1) reduced radiation-dependent activation of a retinoic acid-sensitive protein kinase (ATM) (Figure 1) and (2) reduced radiation-induced ATM-dependent phosphorylation of SP1 (Figure 2). This observation provides experimental evidence for a link between the retinoic acid pathway and a DNA damage response of importance to radiation carcinogenesis. Our observations suggest that deficiency in the retinoic acid pathway, such as loss of ALDH1A1 expression in GDFs from Gorlin syndrome patients, should be considered when predicting or assessing radiation health effects. The significance of these findings is expanded upon in the following sections.

### 4.1. Radiation Carcinogenesis, Retinoic Acid Deficiency and Possible Implications for Secondary Cancers

Retinoic acid deficiency is a common feature associated with cancer patients [29] and its deficiency promotes carcinogenesis in humans and experimental animal models [6,7,8,9]. The deficient expression of ALDH1A1 correlates with deficient activation of a retinoic acid-sensitive kinase (ATM) of importance to radiation carcinogenesis. These observations suggest that retinoic acid-deficiency can plausibly contribute to pathophysiology in radiation-sensitive phenotypes. We have previously discussed the complex linkage between retinoic acid-deficiency and carcinogenesis, which includes alterations in DNA damage repair, cell cycle checkpoint activation, differentiation of the stem cell niche, altered developmental programming and modulation of signal transduction pathways regulating epigenetic carcinogenesis [3]. The Hedgehog pathway is the primary focal point in Gorlin syndrome and experimental evidence highlights a significant intertwined relationship between the retinoic acid and Hedgehog pathways. Specifically, retinoic acid can antagonize the Hedgehog pathway by inducing Patched [30] and there are a number of reports demonstrating that the retinoic acid and Hedgehog pathways interact in developmental biology [31]. Retinoic acid deficiency, therefore, has the potential to eliminate a negative feedback process for the Hedgehog pathway. Deficient ATM activation in GDFs represents a molecular mechanism that is analogous to Ataxia-telangiectasia patients who display genetic inactivation of ATM and are highly susceptible to radiation carcinogenesis [32]. ATM patients display a significant decrease in serum retinol levels [33,34] which is a precursor to retinoic acid. Therefore, ATM and Gorlin syndrome patients share molecular deficits associated with both retinoic acid and ATM pathways.

Secondary cancers remain an unwanted side-effect of radiotherapy [35,36]. By extension, it is feasible that deficits in the retinoid pathway could contribute to secondary cancers induced by radiotherapy. In support of this statement, a subset of cancer patients undergoing radiotherapy will be retinoic acid-deficient [29] and subject to cellular and molecular mechanisms (e.g., deficient ATM activation, reduced stem cell differentiation, loss of tumor suppressor activity) that regulate carcinogenesis in a retinoic acid-sensitive fashion. Retinoic acid-deficiency is not dependent on loss of aldehyde dehydrogenase activity, as observed in cells from Gorlin syndrome patients, and there may be multiple mechanisms by which a retinoic acid-deficient phenotype could arise. Diet, lifestyle and therapeutic pressures can significantly impact retinoic acid levels and specific examples include retinoid depletion by non-ionizing radiation (oxidative and non-oxidative mechanisms) [37,38], cigarette smoke [39] and environmental toxicants [40]. Cigarette smoke in particular produces supra-additive effects on lung cancers associated with radon exposure [41]. Retinoic acid levels are further regulated by P450-mediated metabolism [42] which can change dramatically in response to perturbation. Thus, it is prudent to determine whether prolonged radiotherapy/chemotherapy, dietary or lifestyle choices deplete retinoids, particularly during the course of radiation therapy, as this scenario might promote secondary cancers.

A Ptch^+/−^ mouse has been developed as an experimental model for Gorlin syndrome [43]. Exposure of Ptch^+/−^ mice to ultraviolet or ionizing radiation produces high basal cell carcinoma incidence [44] and treatment of the Ptch^+/−^ mouse with retinoic acid reduces basal cell carcinoma [8]. Due to severe adverse side-effects retinoic acid is not used to manage cancer in Gorlin syndrome patients and alternative retinoid pharmacons (e.g., Tazarotene) with significantly reduced toxicity show promise as alternative therapeutics [8]. Among the pleiotropic effects of retinoids, the regulation of stem cell differentiation has led to important applications in differentiation therapy [30]. It is noteworthy that short term (24 h) retinoic acid treatment *in vitro* can induce human stem cell differentiation [45]. This might enable development of optimal chronic retinoid treatment strategies, such as administration at time intervals that promote differentiation of the cancer stem cell niche without adverse side-effects. Incremental decreases in the cancer stem cell niche over time could result in significant reductions in cancer risk which may be particularly relevant to susceptible populations. Our data suggest that a short-term retinoic acid pulse does not downregulate ATM and therefore, would not be expected to significantly increase cancer risk by mechanisms involving ATM deficits that might occur with extended retinoic acid exposure (Figure 4). Therefore, we believe there is merit in considering alternative treatment schedules for the application of retinoid pharmacons as anti-cancer agents.

### 4.2. DNA Damage Repair and Radiosensitization

We first consider long-term retinoic acid treatment where results from the present study connect with work from independent investigators demonstrating retinoic acid can function as a radiosensitizer [28]. Long-term (5 day) treatment of both NHDFs and GDFs with retinoic acid downregulated ATM protein levels in association with reduced clonogenic survival of irradiated cells (Figure 4). Therefore, the downregulation of ATM by retinoic acid is one mechanism for radiosensitization. There are many examples illustrating that ATM inhibitors dramatically increase radiation cell killing [46,47] and loss of ATM protein would serve the same function. In addition, we have demonstrated that GDFs show reduced clonogenic survival after exposure to X-radiation, as compared with NHDFs [3]. GDFs display impaired ATM activation by radiation (Figure 1), illustrating that multiple mechanisms can lead to deficits in ATM activity and the potential for radiosensitization by this mechanism. It will be interesting to determine whether ATM is down-regulated in other models of retinoic acid radiosensitization. In view of the connection between loss of ATM activity and increased cancer risk, it may also be informative to determine whether ATM protein levels are reduced in models where retinoids increase cancer risk [48].

Studies investigating short-term effects of retinoic acid on ATM activation were designed to validate a role for retinoic acid in ATM activation. Our experimental results showed that the ALDH1A1-deficient phenotype is deficient in ATM activation and that pharmacological retinoic acid supplementation could enhance ATM activation, supporting our major hypothesis. Adverse patient skin reactions to radiotherapy are common (36%–100%) [49] and include pruritus, erythema, edema, desquamation, necrosis, ulceration and/or hemorrhage (collectively referred to as radiation dermatitis or radiation induced skin reaction) [50]. Injury to the skin can be a dose-limiting factor for radiotherapy [51] and may require changes to the person’s radiation schedule (if severe) [52], therefore, managing skin reactions is an important component of radiotherapy. Short-term treatment of GDFs with retinoic acid improved radiation-induced ATM activation (Figure 4B), raising the possibility that short term topical administration of retinoic acid-related pharmacons may improve DNA damage repair in skin and reduce the severity of radiation dermatitis. With continued improvements in the delivery of retinoids to the skin [53] and development of retinoid pharmacons with reduced toxicity [8], topical administration shortly before radiation exposure might selectively protect skin and eliminate systemic effects that could impact tumor response to therapy.

### 4.3. Bulk Chemical Bond Patterns in Live Cells

The IncubATR technology provides an unbiased fingerprint of the biological system in a non-destructive fashion without need for contrast agents. The caveat is that it is presently unclear if a band detected in live cell studies represents the same chemical bond attributed to that band in an IR spectroscopy correlation table. As one example, Figure 5C shows loss of a band in the range of 1656–1658 cm^−1^ (see arrow). This band correlates to membrane-associated helical proteins and our previous proteomics study demonstrated loss of several abundant proteins with prominent helical structures [3]. Thus, future studies will determine whether ectopic expression of these proteins in GDFs can restore the appearance of this band. Alternatively, the chemical bond patterns may represent a more global/integrated representation of the complex system, in which case, gain- and loss-of-function approaches targeting a single protein may only achieve incremental impact on the appearance of bands or be insufficient to reach detectable limits. Thus, our understanding of chemical bond patterns in complex biological systems is in its infancy and our long-term goal is to determine where this new technology exhibits predictive capacity to pave the way for new mechanistic insights of importance to cancer research and therapeutics.

Toward achieving this goal, we demonstrate that FTIR-ATR can discriminate between NHDF and GDF phenotypes in live cell studies, as well as perturbation of GDFs with retinoic acid (Figure 5 and Figure 6). In view of ALDH1A1-deficiency associated with GDFs, our goal was to determine whether chemical bond patterns in GDFs could be reverted or shifted toward the NHDF phenotype with pharmacological retinoic acid supplementation. While GDF chemical bond patterns were modulated by retinoic acid, the shift was not consistent with reversion to the normal phenotype. This may be due to the fact that GDFs exhibit a fundamental baseline change in their phenotype, relative to NHDFs [3,17]. It is plausible that ALDH1A1-/retinoic acid-deficiency could contribute to this altered phenotype through the modulation of developmental stages regulated by retinoic acid, which may not be amenable to reversion by treatment with retinoic acid (e.g., DNA methylation or other stable epigenetic alteration). Alternatively, at the time these studies were conducted we did not yet know that ATM protein levels were downregulated under this condition, which may also contribute to the observed changes in chemical bond patterns. Finally, GDFs show diminished radiation-induced ATM activation (Figure 1) and it may be informative to compare chemical bond patterns in GDFs with the corresponding chemical bond pattern of cells from ATM patients who are also deficient in ATM activity.
